# The factors associated with orthorexia nervosa in type 2 diabetes and their effect on diabetes self-management scores

**DOI:** 10.1007/s40519-023-01552-5

**Published:** 2023-02-21

**Authors:** Hülya Kamarli Altun, Caner Özyildirim, Şeyma Koç, Hatice Nur Aksoy, Beyza Sağir, Merve Sefa Bozkurt, Hakan Karasu

**Affiliations:** Department of Nutrition and Dietetics, Faculty of Health Sciences, Akdeniz University, Dumlupinar Boulevard, Campus, 07058 Antalya, Türkiye

**Keywords:** Type 2 diabetes, Orthorexia nervosa, Diabetes self-management, Diet

## Abstract

**Purpose:**

This study aimed to determine the factors affecting the ORTO-R scores in individuals with T2DM and to investigate their effect on diabetes self-management.

**Methods:**

The study included 373 individuals with type 2 diabetes between the ages of 18–65 who applied to Akdeniz University Hospital Endocrinology and Metabolic Diseases Polyclinic between January and May 2022. A questionnaire including sociodemographic data, information about diabetes, and nutritional habits, and the ORTO-R and Type 2 Diabetes Self-Management Scales were used to collect data. Linear regression analysis was performed to determine the factors affecting ORTO-R.

**Results:**

The linear regression analysis showed that age, gender, education level, and duration of diabetes affected ORTO-R scores in patients with type 2 diabetes. Body mass index, comorbidities (cardiovascular diseases, kidney diseases and hypertension), diabetes-related complications, diabetes treatment method and dieting had no significant contribution to the model (*p* > 0.05). We also found that education level, comorbidities, diabetes-related complications, diabetes treatment method, dieting, and BMI can affect diabetes self-management.

**Conclusion:**

It should be kept in mind that type 2 diabetes are at risk of orthorexia nervosa (ON) in terms of various aspects such as age, gender, education level and duration of diabetes. Since the factors affecting the risk of ON and the factors affecting diabetes self-management are intertwined, orthorexic tendencies should be kept under control while trying to increase self-management in these patients. In this respect, developing individual recommendations according to the psychosocial characteristics of patients may be an effective approach.

**Level of evidence:**

Level V, cross-sectional study.

**Supplementary Information:**

The online version contains supplementary material available at 10.1007/s40519-023-01552-5.

## Introduction

Type 2 diabetes mellitus (T2DM) is a demanding public health problem with a constantly increasing prevalence, causing serious health expenditures [1]. It is estimated that approximately 536 million people in the world have T2DM and this number is expected to reach 783 million in 2045 [2]. Patients with T2DM need to comply with a series of self-care behaviours in order to control the disease and prevent related complications [3]. This approach, called self-management, is accepted as the basic practice to improve metabolic control and quality of life, reduce the risk of complications and health expenditures, along with pharmacological treatment [4–6]. The concept of self-management refers to the level of the patient's ability to adapt to the psychosocial effects, symptoms, treatment processes, and lifestyle changes required by the disease, and to actively participate in the treatment process, especially in chronic diseases. There are seven basic self-care behaviours for patients with diabetes, and these consist of healthy eating, being physically active, blood glucose monitoring, medication adherence, problem-solving skills, coping skills, and risk reduction behaviours [7, 8]. However, some diabetic patients may be excessively attentive to the self-care behaviour of healthy eating, leading to eating disorders.

Orthorexia nervosa (ON) is one of the unclassifiable eating behaviour disorders and is briefly defined as an obsession with healthy eating. ON is not officially recognized as an eating disorder or obsessive–compulsive classification in The Diagnostic and Statistical Manual of Mental Disorders (DSM-5) or the International Statistical Classification of Diseases and Related Health Problems (ICD-10). Although healthy eating habits are not pathological, they can be considered as a disorder when they are exaggerated, prolonged, and when they negatively affect daily life. The difference between ON and healthy eating is that ON has severe restrictive dietary behaviours and strict diet-related self-discipline, adhering to evidence-based or non-evidence-based information to be healthy [9]. In addition, obsessive behaviours associated with  ON are related to the quality of the food consumed rather than the amount [10]. Moreover, individuals with ON spend more time and effort on purchasing, planning, and preparing food to consume healthy and pure meals [11]. The obsessive symptoms seen in ON compels individuals to adopt strict diets or to remove essential nutrients from their diets, and therefore, inadequate, and unbalanced nutrition can occur [12–14]. In recent years, ON has gained increased attention in scientific research [15] and studies have been conducted to determine whether orthorexic behaviours are observed in patients with diabetes mellitus.

Although there are studies investigating the relationship between eating disorders and T2DM [16–19], our knowledge about the relationship between ON and T2DM is limited. When the relationship between chronic diseases and the prevalence of ON was examined, it was found that the highest prevalence was seen in T2DM [20] with 65.5% [21]. Despite this reported high prevalence of ON in patients with T2DM, neither the factors affecting ON in these patients nor their effects on diabetes self-management have been studied. In addition, some factors that affect diabetes self-management are also associated with the risk of ON (age, gender, education level, dieting, BMI, etc.) [15, 22, 23]. In this study, we aimed to determine the factors affecting ORTO-R scores in people with T2DM and to determine their effect on diabetes self-management. In T2DM, compliance with a healthy diet is essential both in maintaining the blood glucose  balance and in preventing future diabetes-related complications. However, we hypothesized that this strict diet plan can increase orthorexic behaviours in people with T2DM.

## Methods

### Study population, design and data collection

This cross-sectional study was carried out in the Endocrinology and Metabolism Outpatient Clinic at Akdeniz University Hospital. The data were collected between January and May 2022. According to hospital records, 811 patients with type 2 diabetes applied to this unit within the past year. Based on the calculation of a sample with a known universe [24] (95% power and 5% margin of error), it was calculated that at least 261 people should be included in the study. Initially, 392 patients with type 2 diabetes were enrolled in the research. Eating disorder diagnosis, bariatric surgery, digestive system disease, psychiatric medication use, neurological disease, pregnancy, and lactation were determined as exclusion criteria. Patients with missing answers in the questionnaire were also excluded (*n* = 19). Thus, the research was completed with 373 patients. The study was conducted in accordance with the Declaration of Helsinki. Akdeniz University Faculty of Medicine Clinical Research Ethics Committee approved all recruitment and assessment procedures (date: 22/12/2021, decision number: KAEK-950).

The questionnaire prepared by the researchers included sociodemographic information, anthropometric measurements, information on health status, blood parameters, ORTO-R Scale and Type 2 Diabetes Self-Management Scale. While the anthropometric measurements were measured by the researchers, the questionnaire was completed by the participants. The fasting blood glucose, glycosylated haemoglobin (HbA1c) and low-density lipoprotein (LDL) cholesterol values were obtained from the hospital record system. In addition, the body mass index (BMI) of all participants was calculated and evaluated using the body weight (kg)/height^2^ (m^2^) formula by taking the height and body weight measurements of the individuals [25].

### Instruments

#### ORTO-R scale

ORTO-R is the revised version of ORTO-15 [26] used to assess the presence of ON. The new scale includes 6 items of ORTO-15 and is scored on a 4-point Likert scale *(never, sometimes, often, and always)* [27]. Higher score on the questionnaire means higher levels of orthorexic behaviours.

The Turkish validity and reliability of the updated ORTO-R version has not been established yet. Therefore, "Reliability Analysis" was used to test the reliability of the scale, "Exploratory Factor Analysis" and "Confirmatory Factor Analysis (DFA)" was performed using the AMOS program to test the construct validity. Before the exploratory factor analysis application, the Kaiser–Meyer–Olkin (KMO) test was applied to test whether the sample size was suitable for factor analysis. As a result of the analysis, it was determined that the use of the ORTO-R was appropriate. Findings on the psychometric properties of the ORTO-R are presented in supplementary tables.

#### Type 2 diabetes self-management scale

This is a five-point Likert type style scale developed by Koc [28] to evaluate the self-management of diabetic patients. The scale consists of a total of 19 questions and three sub-dimensions, namely "Healthy Lifestyle Behaviours" (eleven questions), "Health Services Use" (four questions) and "Blood Glucose Management" (four questions). While evaluating the scale, the “never” response is scored as 1 point, whereas “always” is scored as 5 points. An increase in the score obtained from the scale indicates that individuals with T2DM have higher self-management. With the factor analysis, the explained variance ratio of the scale consisting of 19 items and three dimensions was calculated as 50.019%. Cronbach Alpha coefficient of the scale was calculated as 0.856. In test–retest analysis, it was evaluated that intraclass correlation coefficient ranged between 0.785 and 0.953.

### Data analysis

The data obtained from the study were evaluated in the SPSS 23.0 statistical package program. Qualitative variables were given as numbers (*n*) and percentage (%). The Chi-square test was used to evaluate categorical variables, and the mean and standard deviation values of the quantitative data were calculated. The conformity of quantitative variables to normal distribution was evaluated with the “Kolmogorov–Smirnov” test. Since the variables did not show normal distribution, Mann–Whitney *U* test was used for two groups and Kruskal–Wallis was used for 3 groups or more. The significance level was accepted as *p* < 0.05 in all statistical analyses. The Bonferroni correction was applied in Kruskal–Wallis analysis. Linear regression analysis was performed to examine the effect of variables on the ORTO-R score.

## Results

The mean age of the participants was 57.4 ± 9.5 years and time from T2DM diagnosis was 12.7 ± 9.0 years. The mean HbA1c value was 7.2 ± 1.9. The rate of participants with overweight was 44.8%, and the rate of those with obesity was 32.4%. The mean type 2 diabetes self-management score was 65.6 ± 16.4, and there was no difference between genders (*p* > 0.05). However, men's ORTO-R score (14.5 ± 3.6) was significantly higher than that of women (13.2 ± 3.3) (*p* = 0.001). These results and all descriptive parameters of the participants are given in Supplementary Table 1.

According to the regression analysis, when the significance level corresponding to the F value was considered, the first, second, third and fifth models were found to be statistically significant (*F* = 2.834; *p* < 0.05; Table 1). Considering the beta coefficient value, *t* value and significance level of the independent variable, the ORTO-R score of women is 1.357 units less than men. Age had a statistically significant effect on the ORTO-R score, and as age increases by 1 unit, it causes a decrease of 0.053 on the ORTO-R score. The ORTO-R score of the literate participants was 2.6 units less than the participants with a bachelor's degree or higher. As the duration of diabetes increases by 1 unit; it caused a decrease of 0.060 points on the ORTO-R score (Table 1).

**Table 1:** Factors affecting orthorexic behaviours in patients with type 2 diabetes

Model	Dependent variable	Independent variable	ß	SH	Beta	*t*	*p*	*F*	Model (*p*)	${R}^{2}$	Durbin–Watson
1	ORTO-R scores	Intercept	14.576	0.267	–	54.667	0.000*	13.952	0.000*	0.036	1.926
		Gender: women	− 1.357	0.363	− 0.190	− 3.735	0.000*				
		Gender: men (R)									
2	ORTO-R scores	Intercept	16.912	1.112	–	15.214	0.000*	7.827	0.005*	0.021	1.959
		Age	− 0.053	0.019	− 0.144	− 2.798	0.005*				
3	ORTO-R scores	Intercept	14.600	0.497	–	29.370	0.000*	3.241	0.012*	0.034	1.977
		Literate	− 2.600	0.796	− 0.205	− 3.267	0.001*				
		Primary school	− 0.925	0.572	− 0.128	− 1.616	0.107				
		Secondary school	− 0.538	0.661	− 0.057	− 0.814	0.416				
		High school	− 0.294	0.647	− 0.033	− 0.455	0.649				
		Bachelor and higher (R)									
4	ORTO-R scores	Intercept	14.375	0.397	–	36.207	0.000*	2.273	0.133	0.006	1.938
		Comorbidities: YES	− 0.675	0.448	− 0.078	− 1.508	0.133				
		Comorbidities: NO (R)									
5	ORTO-R scores	Intercept	14.605	0.314	–	46.548	0.000*	8.864	0.003*	0.023	1.961
		Duration of diabetes	− 0.060	0.020	− 0.153	− 2.977	0.003*				
6	ORTO-R scores	Intercept	13.995	0.240	–	58.221	0.000*	0.955	0.329	0.003	1.925
		Diabetes-related complications: YES	− 0.366	0.374	− 0.051	− 0.977	0.329				
		Diabetes-related complications: NO (R)									
7	ORTO-R scores	Intercept	14.333	0.495	–	28.979	0.000*	2.319	0.057	0.025	1.931
		Treatment: diet only	− 0.500	1.133	− 0.025	− 0.441	0.659				
		Treatment: oral antidiabetics	− 0.168	0.569	− 0.023	− 0.295	0.768				
		Treatment: insulin	− 1.333	0.598	− 0.172	− 2.231	0.026				
		Treatment: diet + exercise	− 0.048	0.736	− 0.004	− 0.065	0.948				
		Treatment: combined medical treatment (R)									
8	ORTO-R scores	Intercept	13.667	0.239	–	57.274	0.000*	1.372	0.242	0.004	1.917
		Dieting: YES	0.439	0.375	0.061	1.171	0.242				
		Dieting: NO (R)									
9	ORTO-R scores	Intercept	13.512	0.322	–	42.004	0.000*	2.301	0.077	0.018	1.927
		BMI: lean	− 4.179	2.068		− 2.021	0.044				
		BMI: normal	0.488	0.506	0.057	0.963	0.336				
		BMI: overweight	0.577	0.422	0.081	1.367	0.173				
		BMI: obese (R)									
10	ORTO-R scores	Intercept	12.563	0.757	–	16.587	0.000*	3.044	0.082	0.008	1.911
		Diabetes self-management score	0.020	0.011	0.090	1.745	0.082				

^*^ *p* < 0.05 statistically significant

We found that the increase in education level, the comorbidities (cardiovascular diseases, kidney diseases and hypertension), and the absence of diabetes-related complications positively affect diabetes self-management (Table 2). In addition, diabetes self-management scores differed according to the treatment method. Insulin therapy was associated with worse diabetes self-management compared to oral antidiabetic use, diet, and physical activity, and combined medical therapy. Obesity was also one of the factors that reduced diabetes self-management (Table 2).

**Table 2:** Variables affecting type 2 diabetes self-management scores

Variables	Type 2 diabetes self-management scores		
	Total (*n* = 373)	Men (*n* = 172)	Women (*n* = 201)
Education status			
Literate*	64.3 ± 17.3	76.7 ± 7.2	62.5 ± 17.7
Primary school	61.4 ± 5.5^a,b^	58.4 ± 16.2^c,d^	63.2 ± 14.8
Secondary school	68.3 ± 17.7^a^	68.8 ± 18.4^c^	67.7 ± 17.2
High school	67.0 ± 6.7	65.7 ± 17.7	69.6 ± 14.6
Bachelor	73.3 ± 12.0^b^	75.5 ± 12.7^d^	70.7 ± 11.0
Postgraduate education*	79.0 ± 14.1	79.0 ± 17.3	79
Comorbidities			
Yes	64.1 ± 16.0^a^	63.8 ± 17.8^b^	64.3 ± 14.6^c^
No	71.2 ± 16.7^a^	71.4 ± 15.8^b^	71.0 ± 18.0^c^
Presence of diabetes complications			
Yes	62.4 ± 17.1^a^	61.8 ± 18.4^b^	63.2 ± 15.6
No	67.8 ± 15.5^a^	69.7 ± 15.8^b^	66.6 ± 15.2
Diabetes treatment method			
Oral antidiabetic	66.0 ± 15.6^a,b^	69.5 ± 16.7^f^	63.7 ± 14.4^g,h^
Insulin	58.2 ± 16.0^a,c,d^	56.8 ± 15.7^f,k^	59.9 ± 16.2^m,n^
*Diet only	66.4 ± 20.9	68.5 ± 23.6	63.4 ± 18.7
Diet and physical activity only	78.3 ± 11.7^b,c,e^	77.3 ± 10.8^k^	79.9 ± 13.4^g,m^
Combined medical therapy	69.6 ± 14.0^d,e^	64.8 ± 18.7	71.6 ± 11.2^h,n^
Dieting			
Yes	75.6 ± 12.5^a^	76.8 ± 12.1^b^	74.5 ± 12.9^c^
No	58.9 ± 15.2^a^	57.9 ± 16.6^b^	59.7 ± 14.0^c^
BMI classification			
*Underweight	49.0 ± 8.6	–	49.0 ± 8.6
Normal	67.7 ± 18.4^c^	68.2 ± 17.8	67.2 ± 19.2
Overweight	67.6 ± 16.1^a^	67.5 ± 16.7^b^	67.9 ± 15.2
Obese	61.8 ± 14.6^a,c^	57.7 ± 17.7^b^	63.3 ± 13.1

Within each variable, there are significant differences between groups with the same letter. Mann–Whitney *U* analysis was used for two group comparisons and Kruskal–Wallis analysis was applied for multiple groups. Bonferroni correction was made

*The number of subjects in the literate, postgraduate, underweight, and diet therapy only groups was too small, so they were not included in the analysis

## Discussion

The aim of this study is to determine the factors affecting orthorexic behaviours in patients with T2DM and to determine their effect on diabetes self-management. It is known that patients with T2DM have an increased risk of eating disorders. Although studies are limited, it is reported that the ON is also high in patients with T2DM [20, 29, 30]. We hypothesized that dieting may increase orthorexic behaviours. Patients with T2DM need to make rapid and intense lifestyle changes and keep their blood glucose  under control. Starting and maintaining a lifelong diet, especially due to chronic illness, is complex in many ways for patients [31]. These patients mostly have to plan their meals, be attentive about portioning and track their carbohydrate intake. Strict diet plans and continuous dietary regimes have been reported to be a risk factor for ON [32]. Barbanti et al. [21] found that stricter adherence to Mediterranean diet and nutritional counselling increases orthorexic traits in patients with T2DM. However, our results showed that dieting and treatment methods had no statistically significant effect on ORTO-R scores. These results may be related to the fact that the diet and healthy nutrition recommendations given by dietitians specializing in diabetes do not always lead to pathological results. This concept, referred to as “Healthy Orthorexia”, is inversely related to eating disorder symptomatology and reflects an interest in healthy foods due to health-related concerns [33, 34]. Being healthy seems to be the motivation for healthy orthorexia, but weight control is the main motivation for ON [34]. Considering that obesity rates are high in patients with T2DM, weight control may be the main motivation in these patients, and they may be at risk for ON. However, it is not always easy to distinguish healthy eating motivations that may be beneficial for patients from ON. Another important point is that although dieting increases the ON tendency, it alone does not explain ON [35]. Psychopathological features and maladaptive personality traits should also be considered [35]. Alcohol, tobacco, and drug use, social media use, body dissatisfaction, body image, self-esteem, anxiety, depressive symptoms, health literacy are some of these factors [15, 36, 37].

We found that being male, younger age, decrease in duration of diabetes and increase in education level were associated with an increase in ORTO-R scores. In  contrast, diabetes self-management scores, BMI class, comorbidities, and diabetes-related complications had no effect on ORTO-R scores. Barbanti et al. reported that in people with type 2 diabetes, young, well-educated women with high BMI are at higher risk ON traits [21]. Although there are studies showing that increased BMI is associated with the risk of ON [21, 38], there are also studies that did not report a relationship between BMI and ON [39, 40], which is in line with our finding. Oberle and Lipschuetz [40] showed that ON symptomatology is unrelated to BMI, and it was positively correlated with perceived muscularity and negatively correlated with perceived body fat. Similar to BMI, results regarding gender are also inconsistent [21, 29, 35, 38]. McComb and Mills, in their review of ON-related psychosocial factors, stated that studies on factors such as age, gender, education level, and BMI showed conflicting results and emphasized that the psychometric properties of the methods used in ON assessment were poor [15]. Patients with diabetes have difficulties both in controlling their diabetes and maintaining an ideal body weight [41]. These patients often feel overwhelmed with demand of self-management and find it difficult to keep up with complicated routines [42]. Disordered eating behaviours have been reported to be quite common in young adults with type 2 diabetes receiving insulin therapy [41]. The present study found that the treatment method had no effect on the ORTO-R scores. These conflicting results may be due to the fact that ON is affected by many other risk factors.

Diabetes self-management aims to keep blood glucose and HbA1c levels under control and to avoid long-term complications. However, patients often have difficulty adapting to lifestyle changes, negatively affecting their diabetes self-management [22]. Age, socioeconomic status, and BMI are some of the many factors that affect glycemic control [23]. Our results suggest that higher education level is associated with better diabetes self-management scores. Higher education level may be associated with better diabetes self-management as it is linked to both access to diabetes-related information resources and greater awareness of diabetes-related complications [43]. In addition, it is known that people with higher education levels have higher self-care behaviours [44] and adherence to treatment [45]. Moreover, diabetes education is associated with better glycemic control in people with higher education levels, whereas it is reversed in people with lower education levels [46]. It has been shown that besides education level, duration of diabetes, type of treatment [47] and comorbidities negatively affect glycemic control [48, 49]. Mehravar et al. [50] reported a relationship between low diabetes self-management scores and microvascular complications. Insulin therapy [47, 51] and combination therapy [52] are associated with worse glycemic outcomes. Our findings are in parallel with these results. In our sample, insulin therapy, comorbidities and diabetes-related complications were also linked with worse diabetes self-management. The complexity in the management of the disease may be the reason for these findings. High diabetes-specific medication regimen complexity was associated with poor glycemic control [53] and microvascular complications [54]. According to the Schmidt-Busby et al.’s [55] systematic review, which investigated the contextual factors affecting diabetes self-management, this inadequacy in self-management can be explained by the following reasons: (1) there may be asymmetry, inadequacy or density in the information provided to patients by healthcare professionals; (2) T2DM patients were embarrassed administering insulin outside of the home because they felt uncomfortable and feared being judged; (3) comorbidities add extra burdens as T2DM patients manage many medications and struggle to cope with the many issues related to their condition; and (4) these patients have difficulty in consuming appropriate foods because they cannot see enough support from family and friends, and there is a prejudice against these patients. These social barriers that patients experience is significant because even when the clinical complexity of the disease is not related to poor diabetes control, the social complexity can result in the inadequate control of diabetes [56]. Further, obesity can make diabetes self-management more challenging. The additional burden that comes with T2DM can complicate the management of the disease as people with obesity already suffer from a weight stigma [57]. In addition, higher BMI is associated with worsening glycemic control [58, 59] and the occurrence of T2DM-related complications [60]. Consistent with these, we found that obesity reduces diabetes self-management scores.

## Conclusions

Adherence to a healthy diet is an important part of both effective self-management for patients with prediabetes, type 1 diabetes, T2DM, and first-line preventive treatment for various noncommunicable diseases. Patients with T2DM are obliged to quickly adapt healthy eating behaviours to their lives, and this may negatively affect diabetes self-management and increase orthorexic tendency. We found that the effect of dieting on ORTO-R scores was not statistically significant. Even if dieting did increase orthorexic tendency, we still think it would be important to distinguish ON from healthy orthorexia. However, it should be kept in mind that these patients are at risk of ON from different aspects such as age, gender, education level and duration of diabetes. Since the factors affecting the risk of ON and the factors affecting diabetes self-management are intertwined, orthorexic tendencies should be kept under control while trying to increase self-management in these patients. In this respect, it may be a good approach to develop individual recommendations according to the psychosocial characteristics of patients.

## Strength and limitations

Although it is recognized that ON affects many patients with T2DM, the factors affecting ON and their effect on diabetes self-management are not yet fully known. To the best of our knowledge, this study is the largest-sampled study investigating ON in adults with T2DM in Turkey. However, the study has some limitations. The ORTO-R scale shows orthorexic tendencies rather than diagnosing ON. Therefore, since we do not have a healthy control group, we cannot arrive at a conclusion about whether orthorexic tendency increases in T2DM disease. In addition, since orthorexic tendencies do not always have to be pathological, it may be more useful to distinguish between healthy orthorexia and ON in future studies. ON is affected by various psychological and social factors that were not examined in this study. The cross-sectional nature of the study does not allow determining whether the variables found to be associated with ORTO-R actually cause it.

## What is already known on this subject?

It is known that the risk of eating disorders increases in patients with T2DM. Studies show that a significant proportion of these patients are also affected by ON. Patients with T2DM need to develop self-management behaviours such as acquiring healthy eating habits, medication use, and blood sugar monitoring. It has been determined that these self-management skills also affect eating behaviours and are associated with the risk of ON.

## What this study adds?

In patients with T2DM, age, gender, education level, and duration of diabetes are associated with ON. On the other hand, dieting, BMI, and treatment methods are not associated with ON. However, all these factors affect diabetes self-management.

## Supplementary Information

Below is the link to the electronic supplementary material.

Supplementary file1 (DOCX 35 KB)
