# Supplementary material for: The factors associated with orthorexia nervosa in type 2 diabetes and their effect on diabetes self-management scores
Source: Eat Weight Disord. 2023 Feb 21;28(1):22. doi: 10.1007/s40519-023-01552-5 (PMC9944002; doi:10.1007/s40519-023-01552-5)
Supplement: Supplementary file 1 — Supplementary file1 (DOCX 35 KB) [file 40519_2023_1552_MOESM1_ESM.docx]

**Table 1. General Characteristic of Participants**

| General characteristics | Total (n=373) | | Men (n=172) | | Women (n=201) | | p |
| --- | --- | --- | --- | --- | --- | --- | --- |
| Age (years, mean±SD) | | | | | | | |
|  | 57.4±9.5 | | 57.5±10.0 | | 57.4±9.2 | | >0.05 |
| Diabetes year (SD±SS) | | | | | | | |
|  | 12.7±9.0 | | 12.6±8.5 | | 12.7±9.5 | | >0.05 |
| Blood Parameters (mean±SD) | | | | | | | |
| Fasting blood glucose (mg/dl) | 161.0±56.2 | | 162.5±53.3 | | 159.6±58.8 | | >0.05 |
| HbA1c (%) | 7.2±1.9 | | 7.1±2.1 | | 7.3±1.6 | | 0.040^*^ |
| LDL-C (mg/dl) | 118.8±33.4 | | 128.1±32.8 | | 110.7±32.0 | | <0.001^*^ |
| Intruments (mean±SD) | | | | | | | |
| ORTO-R Scores | 13.8±3.5 | | 14.5±3.6 | | 13.2±3.3 | | 0.001 |
| Type 2 Diabetes Self-Management Scores | 65.6±16.4 | | 65.8±17.5 | | 65.4±15.4 | | >0.05 |
| Education status | | | | | | | |
|  | **n** | **%** | **n** | **%** | **n** | **%** |  |
| Literate | 32 | 8.6 | 4 | 2.3 | 28 | 13.9 | 0.001^*^ |
| Primary school | 154 | 41.3 | 58 | 33.7 | 96 | 47.8 |  |
| Secondary school | 65 | 17.4 | 36 | 20.9 | 29 | 14.4 |  |
| High school | 72 | 19.3 | 47 | 27.3 | 25 | 12.4 |  |
| Bachelor | 46 | 12.3 | 24 | 14.0 | 22 | 10.9 |  |
| Postgraduate education | 4 | 1.1 | 3 | 1.7 | 1 | 0.5 |  |
| Marital status | | | | | | | |
|  | **n** | **%** | **n** | **%** | **n** | **%** |  |
| Married | 320 | 85.8 | 154 | 89.5 | 166 | 82.6 | >0.05 |
| Single | 53 | 14.2 | 18 | 10.5 | 35 | 17.4 |  |
| Smoking | | | | | | | |
|  | **n** | **%** | **n** | **%** | **n** | **%** |  |
| Yes | 57 | 15.3 | 41 | 23.8 | 16 | 8.0 | <0.001^*^ |
| No | 213 | 57.1 | 61 | 35.5 | 152 | 75.6 |  |
| Former smokers | 103 | 27.6 | 70 | 40.7 | 33 | 16.4 |  |
| BMI Classification | | | | | | | |
|  | **n** | **%** | **n** | **%** | **n** | **%** |  |
| Thin | 3 | 0.8 | - | - | 3 | 1.5 | - |
| Normal | 82 | 22.0 | 41 | 23.8 | 41 | 20.4 | >0.05 |
| Overweight | 167 | 44.8 | 98 | 57.0 | 69 | 34.3 | >0.05 |
| Obese | 121 | 32.4 | 33 | 19.2 | 88 | 43.8 | 0.035^*^ |
| Presence of disease except diabetes | | | | | | | |
|  | **n** | **%** | **n** | **%** | **n** | **%** |  |
| Yes | 293 | 78.6 | 127 | 73.8 | 166 | 82.6 | 0.044^*^ |
| No | 80 | 21.4 | 45 | 26.2 | 35 | 17.4 |  |
| Presence of diabetes complications | | | | | | | |
|  | **n** | **%** | **n** | **%** | **n** | **%** |  |
| Yes | 154 | 41.3 | 85 | 49.4 | 69 | 34.3 | 0.004^*^ |
| No | 219 | 58.7 | 87 | 50.6 | 132 | 65.7 |  |
| Family history of diabetes | | | | | | | |
|  | **n** | **%** | **n** | **%** | **n** | **%** |  |
| Yes | 270 | 72.4 | 130 | 75.6 | 140 | 69.7 |  |
| No | 103 | 27.6 | 42 | 24.4 | 61 | 30.3 |  |
| Diabetes treatment method | | | | | | | |
|  | **n** | **%** | **n** | **%** | **n** | **%** |  |
| Oral antidiabetic | 157 | 42.1 | 64 | 37.2 | 93 | 46.3 | 0.002* |
| Insulin | 111 | 29.7 | 60 | 34.9 | 51 | 25.4 |  |
| Diet only | 12 | 3.2 | 7 | 4.1 | 5 | 2.5 |  |
| Diet and physical activity only | 42 | 11.3 | 26 | 15.1 | 16 | 8.0 |  |
| Combined medical therapy | 51 | 13.7 | 15 | 8.7 | 36 | 17.8 |  |

^HbA1c: Glycosylated hemoglobin LDL-C; Low density lipoprotein cholesterol,^

^* Chi-square test was used for categorical variables.^

The Turkish validity and reliability of the updated ORTO-R version has not been studied yet. Therefore, "Reliability Analysis" was used to test the reliability of the scale, while "Exploratory Factor Analysis" and "Confirmatory Factor Analysis (DFA)" were performed using the AMOS program to test the construct validity. As a result of the analysis, it was determined that the Kaiser-Meyer-Olkin Test (KMO) value was 0.662 (Table 2). Values between 0.5 and 1.0 are considered acceptable as KMO values, while values below 0.5 indicate that the factor analysis is not appropriate. In addition, when the results of the Bartlett Sphericity test were examined, it was seen that the chi-square value obtained was acceptable (χ2(15) = 469.624; *p* < 0.05) (Table 2).

**Table 2: Exploratory factor analysis results of ORTO-R**

| Items | Factor 1 | Factor 2 | Total Item Correlations |
| --- | --- | --- | --- |
| Are your rigid and restrictive dietary choices conditioned by your worry about your health status? | 0.715 |  | 0.496 |
| Would you agree that eating healthy food increases your self-esteem? | 0.860 |  | 0.641 |
| Do you believe that strict consuming only of healthy food may improve your appearance? | 0.865 |  | 0.646 |
| In the last three months, did thoughts of food make you feel guilt, ashamed and anxious? |  | 0.820 | 0.452 |
| Does thinking about food excessively worry you for more than three hours a day? |  | 0.798 | 0.419 |
| Does eating healthy food change your lifestyle (frequency of eating out, friends, ...)? |  | 0.591 | 0.331 |
| Reliability | 0.761 | 0.579 | 0.649 |
| Eigenvalue | 2.217 | 1.569 |  |
| Explained Variance | 35.133 | 27.970 | 63.103 |
| KMO =0.662; χ2(15) =469.624; Bartlett Sphericity Test (p) = 0.000 | | | |

^KMO= Kaiser-Meyer-Olkin Test^

In order to reveal the factor pattern of the scale, principal component analysis was chosen as the factorization method, and varimax, one of the vertical rotation methods, was chosen as rotation. In the exploratory factor analysis performed to reveal the factor pattern of the scale, six items were collected in two sub-dimensions. These factors explain 63.13% of the total variance (Table 2). In multifactorial designs, it is considered sufficient if the explained variance is above 50%. When the reliability of the scale and its sub-dimensions were evaluated separately, the reliability coefficients were found to be 0.761 for the first dimension, 0.579 for the second dimension, and 0.649 for the overall scale, and it was found to have good reliability (Table 2). A Cronbach Alpha value greater than 0.50 indicates that the scales used are reliable. This shows that the internal consistency of the scale used in the study is good.

**Table 3: Results regarding the measurement model of ORTO-R**

|  | Items | Factor Loads | Standart Error | t | p |
| --- | --- | --- | --- | --- | --- |
| Factor 1 | Are your rigid and restrictive dietary choices condi- tioned by your worry about your health status? | 0.562 | - | - | - |
|  | Would you agree that eating healthy food increases your self-esteem? | 0.806 | 0.150 | 9.428 | *** |
|  | Do you believe that strict consuming only of healthy food may improve your appearance? | 0.802 | 0.145 | 9.450 | *** |
| Factor 2 | In the last three months, did thoughts of food make you feel guilt, ashamed and anxious? | 0.710 | - | - | - |
|  | Does thinking about food excessively worry you for more than three hours a day? | 0.614 | 0.145 | 5.329 | *** |
|  | Does eating healthy food change your lifestyle (frequency of eating out, friends, ...)? | 0.439 | 0.150 | 5.267 | *** |

When the correlations between the variables are examined, it is seen that the factor loads of the items are above 0.40 and all correlation relationships are significant (Figure 1; Table 3).

**Table 4: Goodness-of-fit values of the structural model of the organizational trust scale**

|  | **Structural Model Values** | **Recommended Values** |
| --- | --- | --- |
| CMIN/DF | 4.639 | ≤5 |
| GFI | 0.968 | ≥0.80 |
| AGFI | 0.917 | ≥0.80 |
| CFI | 0.936 | ≥0.80 |
| TLI | 0.881 | ≥0.80 |
| IFI | 0.937 | ≥0.80 |
| RFI | 0.853 | ≥0.80 |
| NFI | 0.922 | ≥0.80 |
| SRMR | 0.074 | ≤0.10 |

^CMIN/DF: Chi-square fit statistics/degree of freedom, GFI: goodness-of-fit index, AGFI: Adjusted goodness-of-fit index, CFI: Comparative Fit Index, TLI: Trucker-Lewis Index, IFI: the Incremental Fit Index, RFI: the Relative Fit Index, The Normed Fit Index, SRMR: Standardized Root Mean Square Residual^

According to confirmatory factor analysis, it was determined that six items and two sub-dimensions were related to the scale structure. The accepted values for the fit indices are provided in the fit index calculations (Table 4).
